# Functional Roles of Pattern Recognition Receptors That Recognize Virus Nucleic Acids in Human Adipose-Derived Mesenchymal Stem Cells

**DOI:** 10.1155/2016/9872138

**Published:** 2016-12-26

**Authors:** Lili Yu, Yongtao Xu, Fangchao Wang, Can Yang, Guoyan Liu, Xiangfeng Song

**Affiliations:** ^1^School of Basic Medical Sciences, Xinxiang Medical University, Xinxiang, Henan 453003, China; ^2^Henan Collaborative Innovation Center of Molecular Diagnosis and Laboratory Medicine, Xinxiang, Henan 453003, China; ^3^School of Biomedical Engineering, Xinxiang Medical University, Xinxiang, Henan 453003, China

## Abstract

Human adipose-derived mesenchymal stem cells (hAD-MSCs) are mesenchymal stem cells with the capability to modulate immune responses. Evidence showing that hAD-MSCs could mediate innate immune responses through pattern recognition receptors (PRRs) is increasing. However, the roles of PRRs in regulating the innate sensing of virus nucleic acids (RNA and DNA) in hAD-MSCs have not yet been investigated. This study focused on the abundant expression of PRRs, including Toll-like receptor 3 (TLR3) and retinoic acid-inducible gene I (RIG-I), which recognize viral RNA, and gamma-interferon inducible protein 16 (IFI16), which recognizes viral DNA in hAD-MSCs. Poly(I:C), a synthetic dsRNA analogy, activated TLR3 and RIG-I and induced the expression of type I interferons (IFN-*α*/*β*) and antivirus proteins, including IFN-stimulating gene 15, 2′5′-oligoadenylate synthetase, and Mx GTPase 1 in hAD-MSCs, which were attenuated by the knockdown of each TLR3 or RIG-I. Synthetic herpes simplex viral DNA (HSV60) activated IFI16 and induced the expression of IFN-*α*/*β* and antivirus proteins in hAD-MSCs, which were inhibited by the knockdown of IFI16. Both poly(I:C) and HSV60 induced the expression of IFN-*α*/*β* through the phosphorylation of IFN-regulatory factor 3. All these results indicated that PRRs recognizing virus nucleic acids were expressed and can mediate antivirus responses in hAD-MSCs.

## 1. Introduction

Mesenchymal stem cells (MSCs) are multipotent adult stem cells capable of differentiation to many cell types, such as adipocytes, osteoblasts, chondrocytes, tendinocytes, myocytes [1]. MSCs can reportedly be isolated from different organs and functions as a source of cells for replacement and regeneration [2]. Adipose tissue is a potential source of MSCs referred to as human adipose-derived MSCs (hAD-MSCs), which have been described as fibroblast-like adherent and multipotent cells [3–5].

Apart from modulating differentiation potential [4], hAD-MSCs can also modulate immune response [6]. Evidence showing that hAD-MSCs possess immunosuppressive properties is increasing [7, 8]. They could inhibit the activation, proliferation, and function of immune cells, including T cells, B cells, and natural killer cells through cell contact [9]. Meanwhile, increasing studies apparently indicate that hAD-MSCs could enhance innate immunity through pattern recognition receptors (PRRs) to secrete proinflammatory cytokines, such as tumor necrosis factor- (TNF-) *α*, interleukin- (IL-) 6, and IL-8 [2, 3, 10–12].

PRR-mediated innate immune response constructs the first line of defense against invading microbes [13, 14]. PRRs recognize highly conserved molecular patterns of microbial pathogens, known as pathogen-associated molecular patterns (PAMPs), and trigger innate immune response against microbes. Several families of PRRs have been identified, including Toll-like receptors (TLRs), retinoic acid-inducible gene I-like receptors (RLRs), nucleotide binding domain-like receptors (NLRs), absent in melanoma-like receptors (ALRs), and DNA sensors [15–18].

Viral PAMPs of nucleic acids, including RNA and DNA, can be detected by different PRRs [19, 20]. TLR3, TLR7, and TLR8 of TLRs and melanoma differentiation-associated factor 5 (MDA5) and RIG-I of RLRs recognize diverse viral RNA. TLR7 and TLR8 recognize viral signal-strand RNA (ssRNA) [15]. TLR3, RIG-I, and MDA5 recognize double-stranded RNA (dsRNA), which can be generated by many types of viruses during replication, and can further be activated by polyinosinic-polycytidylic acid [poly(I:C)], a synthetic dsRNA analog [15, 16]. TLR3 uses the TIR-domain-containing adapter-inducing interferon-*β* (TRIF) as the adaptor to induce the downstream signaling [21]. RIG-I and MDA5 trigger the signaling pathway using IFN-*β* promoter stimulator (IPS) as an adaptor [22].

The DNA of virus is mainly detected by PRRs of TLR9 and cytosolic DNA sensors [23, 24]. TLR9 was identified as the recognition of CpG DNA. Cytosolic DNA sensor recognizes viral genomic DNA, including DNA-dependent activator of interferon (IFN) regulatory factor (DAI), RNA polymerase III (polyIII), gamma-interferon-inducible protein (IFI16), and cyclic GMP-AMP synthase (cGAS) [25]. The DAI was initially discovered as a cytosolic DNA sensor initiating innate immune responses [26]. cGAS can bind to microbial DNA and be identified as a cytosolic DNA sensor [27]. polyIII can be considered a DNA sensor because this polymerase transcribes DNA to RNA and activates RIG-I [28]. IFI16 is a DNA-binding protein that mediates DNA virus-triggered innate antiviral response signaling [29]. The stimulator of IFN genes (STING) is a common adaptor of DNA sensor-initiated signaling [30].

Both viral RNA and DNA recognition pathways of PRRs convert the activation of IFN-regulatory factor (IRF), thereby inducing the expression of type I interferons, IFN-*α*, and IFN-*β* [31]. Type I interferon production is a primary antiviral response [31]. The elimination of virus by IFN-*α* and IFN-*β* aims to induce antiviral protein synthesis in the infected cells and activate adaptive immunity system [32]. Several antiviral proteins, including IFN-stimulated gene (ISG15), 2′5′-oligoadenylate synthetase 1 (OAS1), and Mx GTPase 1 (Mx1), have been found [33–35]. These antiviral proteins individually amplify antiviral signaling, degrade viral RNA, inhibit viral protein translocation, and block viral mRNA transcription.

Recent studies have shown that hAD-MSCs express virtually all TLRs, except TLR8 and demonstrated that hAD-MSCs possess active and functional TLR2, TLR3, and TLR4, which can be activated by their agonists and can trigger downstream signaling events [11, 36]. However, the PRRs which recognize nucleic acids have not been studied. In this study, we demonstrated that hAD-MSCs highly expressed PRRs that recognized nucleic acids, including TLR3, RIG-I, and IFI16. The poly(I:C), a synthetic dsRNA, activating TLR3 and RIG-I, and HSV60, a synthetic fragment of DNA sequence of herpes simplex virus (HSV), activating IFI16, induced innate immune responses through the expression of type I interferons and antiviral proteins in hAD-MSCs. The present study indicates the functional roles of PRRs recognized virus nucleic acids, which may provide a better understanding of hAD-MSCs response to viral infection.

## 2. Results

### 2.1. Expression of PRRs of Sensing Viral Nucleic Acids in hAD-MSCs

**Figure 1:**
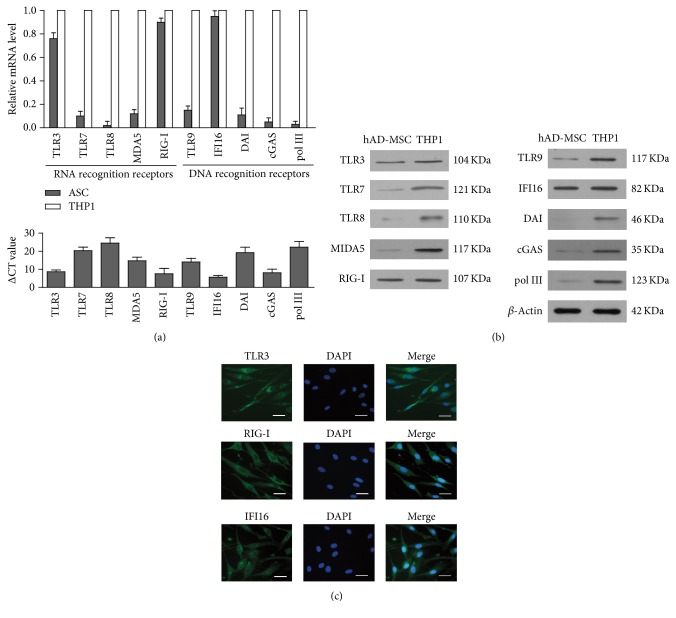
Expression of PRRs recognized viral RNA and DNA. (a) Total RNA was extracted from human adipose-derived mesenchymal stem cells (hAD-MSCs) and THP1 cell lines (THP1). Relative mRNA levels of PRRs recognized viral RNA nucleic acids, including TLR3, TLR7, TLR8, MDA5, and RIG-I, and RPRs recognized viral DNA nucleic acids, including TLR9, IFI16, DAI, cGAS, and polIII, were examined using real-time PCR by normalizing to GAPDH in the upper panel. The ΔCt values of genes expression in hAD-MSCs were shown in the lower panel. (b) The protein level of PRRs recognized viral nucleic acids in hAD-MSCs and THP1 were determined by Western blot using specific antibodies. *β*-Actin was used as loading control. (c) Distribution of TLR3, RIG-I, and IFI16. Indirect immunofluorescence staining using specific antibody, respectively, against TLR3, RIG-I, and IFI16 was performed in hAD-MSCs (left panels). The cells were staining with DAPI (middle panels). Images of TLR3, RIG-I, and IFI16 and DAPI staining were merged, respectively (right panels). Data are present as the mean ± SEM of three experiments. Images represent at least three experiments. Scale bar = 20 *μ*m.

To determine the expression of PRRs of sensing viral nucleic acids, we analyzed the mRNA levels of PRRs recognizing viral RNA and DNA sensors in hAD-MSCs, according to real-time PCR. The results showed that PRRs that recognized viral RNA, including TLR3 and RIG-I, as well as DNA recognition receptor IFI16, were highly expressed at a comparable level in hAD-MSCs, compared with ThP1 cells, a monocytic human cell line used as a positive control (Figure 1(a)). By contrast, other PRRs, such as TLR7, TLR8, MDA5, TLR9, DAI, cGAS, and polyIII, were expressed in low level. The expression of virus RNA sensors and DNA sensors in hAD-MSCs at protein levels were confirmed using Western blot (Figure 1(b)). Indirect immunofluorescence staining showed that the localization of RIG-I and TLR3 was expressed in cytoplasm, and IFI16 was expressed in both the cytoplasm and nucleus (Figure 1(c)).

### 2.2. Poly(I:C) Induced the Expression of Type I Interferons and Antiviral Proteins in hAD-MSCs

**Figure 2:**
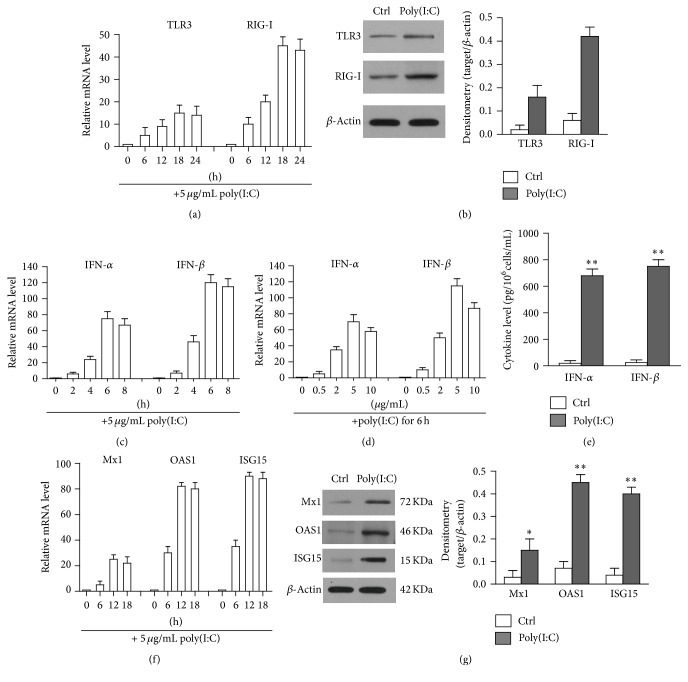
Poly(I:C)-induced immune responses. (a) Upregulation of TLR3 and RIG-I. hAD-MSCs were stimulated with 5 *μ*g/mL poly(I:C) at indicated time. Relative mRNA levels of TLR3 and RIG-I were determined by real-time RCR at different time points. (b) The protein levels of TLR3 and RIG-I were determined by Western blot. hAD-MSCs were lysed 24 h after poly(I:C) stimulation. *β*-Actin was used as loading controls. (c) Poly(I:C) induced the expression of IFN-*α* and IFN-*β* in a time-dependent manner. Total RNA was extracted from hAD-MSCs, which were stimulated 5 *μ*g/mL poly(I:C) in different time. Relative mRNA levels of IFN-*α* and IFN-*β* were determined using real-time PCR by normalizing to *β*-actin. (d) Poly(I:C) induced the expression of IFN-*α* and IFN-*β* in a dose-dependent manner. Total RNA were stimulated with the indicated dose of poly(I:C) for 6 h. Relative mRNA levels of IFN-*α* and IFN-*β* were determined by real-time PCR. (e) The secretion of IFN-*α* and IFN-*β*. hAD-MSCs were stimulated with poly(I:C). The expression levels of IFN-*α* and IFN-*β* in culture medium were measured by ELISA. (f) Expression of antiviral proteins in mRNA level. Total RNA was extracted from hAD-MSCs at the different time points after poly(I:C) stimulation. Relative mRNA levels of Mx1, OAS1, and ISG15 were determined using real-time PCR. (g) Expression of antiviral proteins in protein levels. The cell lysates of hAD-MSCs were to probe antiviral proteins by Western blot using specific antibodies after stimulation with poly(I:C) 24 h. The cells treated with LyoVec along served as control (Ctrl). Western blot images are representatives of at least three experiments. Data are presented as the mean ± SEM of three experiments. ^*∗*^ *P* < 0.05; ^*∗∗*^ *P* < 0.01.

We found that TLR3 and RIG-I were highly expressed in hAD-MSCs. They recognized double-stranded RNA and induced immune response in different cells [37, 38]. Poly(I:C) was the synthetic double-stranded RNA and mimics the virus infection of the cells. Therefore, we investigated the function of TLR3 and RIG-I by stimulating hAD-MSCs with poly(I:C). As shown in Figure 2(a), poly(I:C) induced the upregulation of TLR3 and RIG-I in a time-dependent manner, and the mRNA level was peaked at 18 h. A 15-fold upregulation in the mRNA level of TLR3 was observed 18 h after poly(I:C) stimulation. The mRNA level of RIG-I was upregulated by over 40-fold. After a 24-hour stimulation, the protein level of RIG-I and TLR3 was identified using Western blot (Figure 2(b)). Real-time PCR results indicate the poly(I:C)-induced expression of IFN-*α* and IFN-*β* at mRNA levels in a time-dependent manner, and the peak mRNA level of IFN-*α* and IFN-*β* appeared at 6 h after poly(I:C) stimulation (Figure 2(c)). Real-time PCR results further indicate that poly(I:C) induced the expression of IFN-*α* and IFN-*β* in a dose-dependent manner, and the plateau mRNA levels were detected with 5 *μ*g/mL poly(I:C) (Figure 2(d)). The protein levels of IFN-*α* and IFN-*β* in culture media were measured using ELISA at 24 h after poly(I:C) stimulation. Furthermore, the results indicated that IFN-*α* and IFN-*β* concentration was significantly increased (Figure 2(e)). We further examined the expression of major antiviral proteins, including Mx1, OAS1, and ISG15 in hAD-MSCs. After poly(I:C) stimulation, the mRNA levels of the antiviral proteins were remarkably upregulated in a time-dependent manner, and the plateau was peaked at 16 h (Figure 2(f)). The upregulation of protein levels was identified using Western blot (Figure 2(g)). In general, poly(I:C) can induce the expression of IFN-*α*/IFN-*β* and antivirus proteins in hAD-MSCs.

### 2.3. Involvement of TLR3 and RIG-I in Poly(I:C)-Induced Innate Antivirus Response

**Figure 3:**
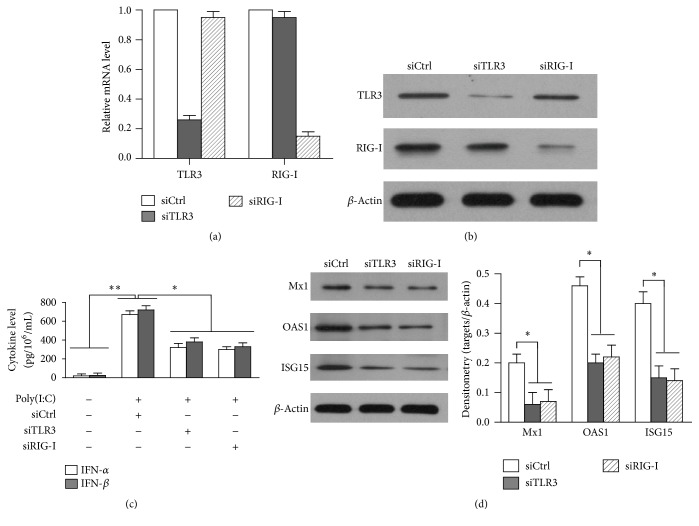
Involvement of TLR3 and RIG-I in poly(I:C)-triggered immune responses. (a) Knockdown of TLR3 or RIG-I at the mRNA levels. hAD-MSCs were transfected with individual siRNAs targeting a scrambled sequence (SiCtrl), TLR3 (siTLR3), and RIG-I (siRIG-I) and the mRNA levels were determined using real-time PCR. (b) Knockdown of TLR3 or RIG-I at the protein levels. hAD-MSCs were transfected with siCtrl, siTLR3, and siRIG-I. After 24 h, the expression of TLR3 and RIG-I was detected by Western blot. (c) IFN-*α* and IFN-*β* secretion. hAD-MSCs were transfected with each siRNA. After 24 h, the cells were stimulated with poly(I:C) and the cytokine levels of IFN-*α* and IFN-*β* in media were measured using ELISA. (d) Expression of antiviral proteins. hAD-MSCs were treated as (c). Antiviral protein levels were determined using Western blot. Western blot images represent at least three experiments. Data are the means ± SEM of three experiments. ^*∗*^ *P* < 0.05; ^*∗∗*^ *P* < 0.01.

To analyze the roles of TLR3 and RIG-I signaling in mediating poly(I:C)-induced immune response in hAD-MSCs, TLR3 and RIG-I silence used each specific small interfering RNA (siRNA), including siTLR3 and siRIG-I. A siRNA targeting a scrambled sequence was used as control (siCtrl). After transfecting with siRNA, the expression of TLR3 and RIG-I at mRNA level (Figure 3(a)) and protein level (Figure 3(b)) was successfully downregulated. Each siRNA reduced >75% of target. Then, the cells were stimulated with poly(I:C). The siRNA targeting individual TLR3 and RIG-I significantly decreased poly(I:C)-induced secretion of IFN-*α* and IFN-*β*, compared with siCtrl (Figure 3(c)). Moreover, poly(I:C)-induced expression of ISG15, OAS1, and Mx1 was significantly reduced by siTLR3 and siRIG-I (Figure 3(d)). The results indicate the involvement of both TLR3 and RIG-I in mediating the poly(I:C)-induced immune responses.

### 2.4. HSV60 Induced the Expression of Type I Interferons and Antiviral Proteins in hAD-MSCs

**Figure 4:**
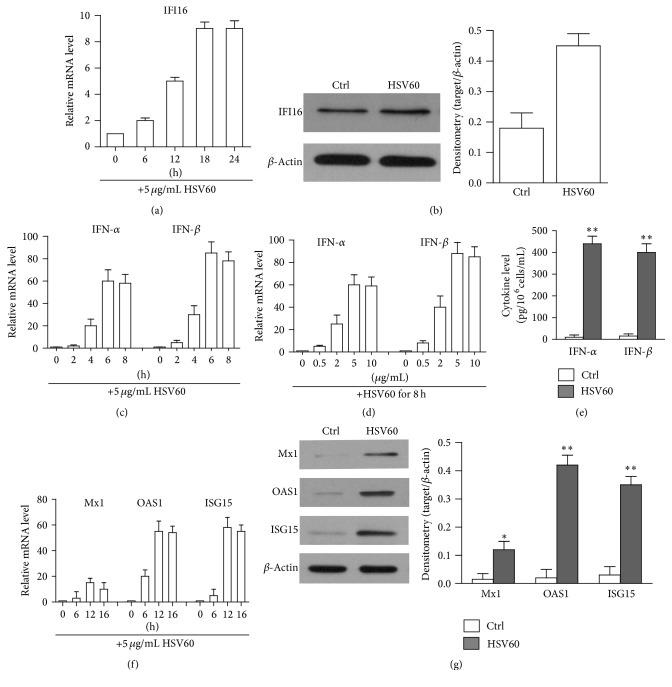
HSV60-induced immune responses. (a) Upregulation of IFI16. hAD-MSCs were stimulated with 5 *μ*g/mL HSV60 at indicated time points. Relative mRNA levels of IFI16 were determined by real-time PCR. (b) The protein levels of IFI16 were determined by Western blot. hAD-MSCs were lysed 24 h after HSV60 stimulation. *β*-Actin was used as loading control. (c) Time-dependent IFN-*α* and IFN-*β* expression. Total mRNA was extracted from hAD-MSCs at the indicated time points post HSV60 stimulation. Relative mRNA levels of IFN-*α* and IFN-*β* were determined using real-time PCR by normalizing to *β*-actin. (d) Dose-dependent IFN-*α* and IFN-*β* expression. hAD-MSCs were stimulated with indicated dose of HSV60 for 6 h. Relative mRNA levels of IFN-*α* and IFN-*β* were determined by real-time PCR. (e) IFN-*α* and IFN-*β* secretion. hAD-MSCs were stimulated with HSV60. After 24 h, IFN-*α* and IFN-*β* levels in culture medium were measured using ELISA. (f) Expression of antiviral proteins in mRNA levels after HSV60 stimulation. Total RNA was extracted from hAD-MSCs at the different times points after stimulation with HSV60. Relative mRNA levels of ISG15, OAS1, and Mx1 were determined using real-time PCR. (g) Expression of antiviral proteins in protein levels. hAD-MSCs were stimulated with HSV60. After 24 h, the cell lysates were extracted to Western blot to probe antiviral proteins. The cells treated with LyoVec alone served as Ctrl. Western blot images represent at least three experiments. Data are presented as the mean ± SEM of three experiments. ^*∗*^ *P* < 0.05; ^*∗∗*^ *P* < 0.01.

Apart from TLR3 and RIG-I, which recognized viral RNA, the IFI16 recognizing viral DNA was further highly expressed in hAD-MSCs compared with other cytosolic DNA sensors (Figures 1(a) and 1(b)). HSV60 was the synthetic fragment of HSV DNA and triggered innate immune responses through IFI16 signaling [39]. Accordingly, we stimulated the hAD-MSCs with HSV60. As shown in Figure 4(a), HSV60 induces the upregulation of IFI16 in a time-dependent manner. After 18 h stimulation, the IFI16 was upregulated by 20-fold. Western blot identified the protein level of IFI16 (Figure 4(b)). HSV60 further induced the expression of IFN-*α* and IFN-*β* at mRNA levels in a time-dependent manner, and the expression peaked at 6 h (Figure 4(c)). After stimulation, the upregulation of IFN-*α* and IFN-*β* expression is in a dose-dependent manner as well, and the plateau mRNAs were detected with 5 *μ*g/mL of HSV60 (Figure 4(d)). ELISA results showed that IFN-*α* and IFN-*β* concentrations in the culture media significantly increased after a 24-h HSV60 stimulation (Figure 4(e)). We further examined the expression of the antivirus proteins. Real-time PCR results showed that HSV60 dramatically upregulated ISG15, OAS1, and Mx1 (Figure 4(f)). The expression pattern of the antiviral protein was further confirmed in the protein level using Western blot (Figure 4(g)). In summary, HSV60 could induce the expression of IFN-*α*/IFN-*β* and antivirus proteins in hAD-MSCs.

### 2.5. Involvement of IFI16 in HSV60-Induced Innate Antivirus Responses

**Figure 5:**
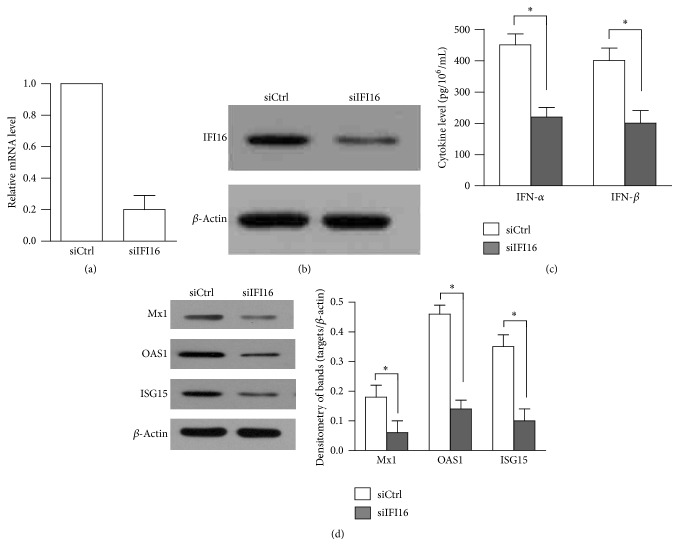
Involvement of IFI16 in HSV60-triggered immune responses. (a) Knockdown of IFI16 at mRNA level. hAD-MSCs were transfected with individual siIFI16 and siCtrl. After 24 h, the expression of IFI16 was determined using real-time PCR. (b) The protein level of knockdown of IFI16. hAD-MSCs were treated as (a) and the expression of IFI16 was determined by Western blot. (c) IFN-*α* and IFN-*β* secretion. hAD-MSCs were transfected with each siRNA. After 24 h, the cells were stimulated with poly(I:C) or HSV60 and the cytokine levels of IFN-*α* and IFN-*β* in media were measured using ELISA. (d) The expression of antiviral proteins. hAD-MSCs were treated as (c). Antiviral protein levels were determined using Western blot. Western blot images represent at least three experiments. Data are the means ± SEM of three experiments. ^*∗*^ *P* < 0.05.

To determine the involvement of IFI16 signaling in HSV60-induced immune responses, the siIFI16 was used for the knockdown of IFI16. The expression of IFI16 at mRNA level (Figure 5(a)) and protein level (Figure 5(b)) was significantly reduced after 24 h siIFI16 transfection compared with the siCtrl. At 24 h after siRNA transfection, the cells were transfected with HSV60. IFN-*α* and IFN-*β* secretions were significantly suppressed when the cells were transfected with siIFI16 compared with siCtrl at 24 h after HSV60 stimulation (Figure 5(c)). Moreover, HSV60 induced expression of antiviral protein levels was significantly decreased by siIFI16 (Figure 5(d)). These results notably indicate the involvement of IFI16 in mediating HSV60-induced immune responses.

### 2.6. Phosphorylation and Translocation of IRF3

**Figure 6:**
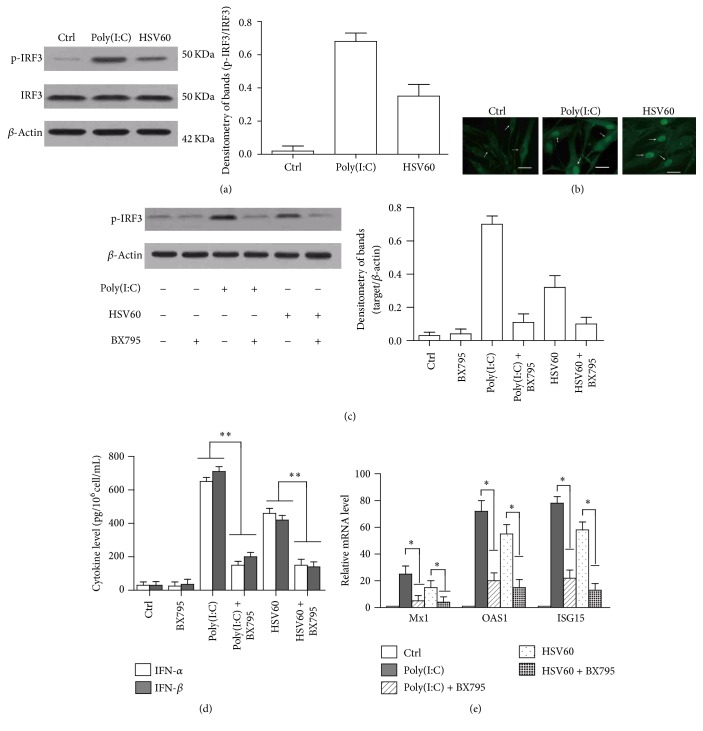
Poly(I:C) and HSV60-induced immune responses through IRF3 activation. (a) Phosphorylation of IRF3 in hAD-MSCs. hAD-MSCs were stimulated with poly(I:C) or HSV60 for 2 h. Cell lysates were analyzed by Western blot to probe phospho-IRF3 (p-IRF3) and total IRF3. *β*-Actin was used as loading control. (b) Nuclear translocation of IRF3. hAD-MSCs were stimulated with poly(I:C) (middle panel) or HSV60 for 4 h. Immunofluorescence staining was performed by IRF3 antibody. Arrows show the representatives of nucleus in hAD-MSCs. (c) Inhibition of IRF3 activation. hAD-MSCs were stimulated with poly(I:C) or HSV60 alone or with poly(I:C) or HSV60 after 2 h before incubation with 1 *μ*g/mL BX795 (an inhibitor of IRF3 activation). At 2 h after poly(I:C) or HSV60 stimulation, p-IRF3 and total IRF3 level were determined by Western blot. (d) IFN-*α* and IFN-*β* secretion. The cells were treated as (c). At 24 h after poly(I:C) or HSV60 stimulation, the cytokine levels in media were measured using ELISA. (e) Expression of antivirus proteins. The cells were treated as (c). At 6 h after poly(I:C) or HSV60 stimulation, the mRNA levels of antiviral proteins, ISG15, OAS1, and Mx1, were determined using real-time PCR. The cells that were treated with LyoVec alone were served as Ctrl. Images represent at least three experiments. Scale bar = 20 *μ*m. Data are the means ± SEM of three experiments. ^*∗*^ *P* < 0.05; ^*∗∗*^ *P* < 0.01.

TLR3, RIG-I, and IFI16 use different adaptors to induce the downstream signaling pathway, but all of the signaling induces the type I interferons through the phosphorylation of IRF3 [21]. Western blot results showed that either poly(I:C) or HSV60 stimulation induced the phosphorylation of IRF3 in the hAD-MSCs (Figure 6(a)). Activated IRF3 must be translocated from the cytoplasm into the cell nucleus to induce cytokine expression. Indirect immunofluorescence staining showed that poly(I:C) or HSV60 stimulation efficiently induced the translocation of IRF3 (Figure 6(b)).

IRF3 phosphorylation could be inhibited by BX795, which is the inhibitor of IRF3 activation.  Figure 6(c) shows that BX795 significantly inhibited poly(I:C), or HSV60 induced the IRF3 phosphorylation in hAD-MSCs. BX795 stimulation alone does not induce the phosphorylation of the IRF3 (Figure 6(c)). BX795 further inhibited the poly(I:C), or HSV60 induced the secretion of IFN-*α* and IFN-*β* (Figure 6(d)), as well as the expression of antiviral proteins, including ISG15, OAS1, and Mx1 (Figure 6(e)). These results indicated that both poly(I:C) and HSV60 induced the expression of type I interferons through IRF3 activation in hAD-MSCs.

## 3. Discussion

PRR-mediated innate immune responses construct the first line of defense against invading microbes [14, 40]. The role of TLRs and NLRs in hAD-MSCs has been studied whereas other PRRs still await investigation [36, 41–44]. Several clinical investigations reveal the susceptibility of hAD-MSCs to different viruses. The main PRRs, including TLR3, TLR7, TLR8, TLR9, MDA5, and RIG-I, recognized virus RNA nucleic acids; and TLR9, cGAS, IFI16, and DAI recognized virus DNA nucleic acids initiate antivirus responses [16, 18, 45]. In the present study, we examined the expression profiles for main PRRs detecting viral RNA and DNA nucleic acids in hAD-MSCs. We found that TLR3, RIG-I, and IFI16 were highly expressed in hAD-MSCs. Other studies further indicated the expression of RIG-I and MDA5 in mesenchymal stem cells from a mouse bone marrow of tibia and femur (mBM-MSCs) [46]. However, we found the low level expression of MDA5 in hAD-MSC compared with mBM-MSCs, possibly because of the source of different tissue sources.

To determine of the functional role of PRRs of the virus nucleic acids detectors in hAD-MSCs, we use poly(I:C) (agonist for TLR3 and RIG-I) [47] and HSV60 (agonist of IFI16) [39] in stimulating hAD-MSCs to initiate the immune responses. The synthetic poly(I:C) represents dsRNA that can be generated by a different type of virus during replication and activated TLR3 and RIG-I. Furthermore, other reports have indicated that poly(I:C) increased the expression of inflammatory cytokine, including TNF*α*, IL-12, and IL-6, not referring to the expression of type I interferons in hAD-MSCs [11]. In this study, we focus on the expression of IFN-*α* and IFN-*β* secretion. Poly(I:C) can actually promote the expression of IFN-*β* in MSCs according to the previous studies [46, 48–50]. The HSV60, a synthetic analogy of HSV genomic DNA, successfully triggered innate immune responses through the IFI16 signaling pathway [39]. In our study, poly(I:C) or HSV60 stimulation induced the upregulation of IFN-*α* and IFN-*β*, which are pleiotropic cytokines against viruses by inducing the expression of antiviral proteins [51, 52]. The best-characterized antiviral proteins are ISG15, OAS1, and Mx1, which inhibit viral replication at multiple levels within the infected cells [53]. We showed that ISG15, Mx1, and OAS1 were dramatically upregulated in hAD-MSCs after stimulating with poly(I:C) or HSV60.

To directly prove the involvement of TLR3 and RIG-I in poly(I:C)-induced antiviral response, we knocked down TLR3 and RIG-I in hAD-MSCs. The specific siRNA for TLR3 and RIG-I significantly decreased poly(I:C)-induced type I interferons and antiviral proteins. These results suggest that both TLR3 and RIG-I all mediated innate antivirus responses in hAD-MSCs, which were reported in other cell types [54, 55]. Furthermore, we prove that IFI16 is involved in the HSV60-induced immune response, which plays an important role in DAN recognition [56]. The specific siRNA for IFI16 significantly decreased HSV60-induced type I interferons and antiviral proteins. Whether the knockdown of TLR3/RIG-I or IFI16 did not fully inhibit the poly(I:C) or HSV60-induced antiviral reaction, these results indicated the possible involvement of other PRRs in the immune response in hAD-MSCs. Our results indicate that TLR3, RIG-I, and IFI16 mediating signaling pathways lead to IRF3 activation in hAD-MSCs, thereby inducing the expression of IFN-*α* and IFN-*β*. Furthermore, BX795 obviously inhibits the pathway and downregulation of the expression of type I interferons and antiviral proteins.

hAD-MSCs are nonhematopoietic multiple progenitor cells found in adipose tissues [57]. They are characterized by their ability for rapid growth and maintenance of their differentiation potential in vivo and vitro [58]. Our work indicates the immunomodulation activity of hAD-MSCs. A growing body of evidence shows that hAD-MSCs expressed the pattern recognition receptors. Our results showed the virus recognition receptors RIG-I, TLR3, and IFI16, which possibly be the mechanism that protects MSCs themselves against virus infection. In other way, the secretion of inflammatory factors and interferon could induce the immune response through the activation of immune cells, which possibly is the compartment part of the antiviral response of whole organisms. Current evidences support the utilization of hAD-MSCs for the treatment of numerous diseases. hAD-MSCs secrete a wide variety of pro- and anti-inflammatory factors and have the potential to affect multiprocesses. Studying differently emerging of PRRs allow us to see the following: the finding raised the possibility that, after virus infection, the cultured hAD-MSCs result in increased expression of PRRs. Clinical study should pay attention to the cultured hAD-MSCs avoiding the virus infection and furthermore transplanted cells from the virus-infected patients require further identification.

Taken together, our findings demonstrate that hAD-MSCs expressed functional virus RNA sensors RIG-I and TLR3 and virus DNA sensor IFI16 in hAD-MSCs. Activation of hAD-MSCs with poly(I:C) and HSV60 triggered the expression of type I interferons and antiviral proteins. The finding of functional PRRs recognized viral nucleic acids currently provides a better understanding of hAD-MSCs responses to viral infection.

## 4. Materials and Methods

### 4.1. Cell Culture

hAD-MSCs were purchased from the Sciencell Research Laboratories (#7510, San Diego, CA, USA). The cells are characterized by immunofluorescence with antibodies specific to CD73, CD90, and CD105, according to the company's instructions. The hAD-MSCs were cultured in the MSCs medium (#7501, Sciencell Research Laboratories), and passage three was used for the following experiments.

THP1 cells were obtained from the China Infrastructure of Cell Line Resources (Beijing, China), which were cultured in RPMI-1640 (11875-093, Life Technologies, Grand Island, NY, USA), supplemented with 10% fetal bovine serum (10099-141, Life Technologies), 100 U/mL penicillin, and 100 mg/mL streptomycin.

### 4.2. Antibodies and Major Reagents

Anti-IFI16 (SAB1408587), anti-DAI (PRS4401), and anti-cGAS (SAB3500110) antibodies were purchased from Sigma (St. Louis, MS, USA). Anti-phospho-IRF3 (number 4947) and anti-ISG15 (number 2743) antibodies were purchased from Cell Signaling Technology (Beverly, MA, USA). Anti-*β*-actin (sc-81178), anti-IRF3 (sc-9082), and anti-Mx1 (sc-50509) antibodies were purchased from Santa Cruz Biotechnology (Santa Cruz, CA, USA). Anti-TLR3 (ab137722), anti-TLR7 (ab45371), anti-TLR8 (ab24185), anti-TLR9 (ab12121), anti-MDA5 (ab69983), anti-RIG-I (ab45428), anti-OAS1 (ab86343), and anti-Pol III (ab22236) antibodies were purchased from Abcam (Cambridge, UK). Poly(I:C)/Lyovec™ (tlrl-piclv), HSV60/Lyovec (tlrl-hsv60c), and BX795 (tlrl-bx7) were purchased from InvivoGen (San Diego, CA, USA). Small interfering RNA (siRNA) targeting RIG-I (sc-61480), TLR3 (sc036685), and IFI16 (sc-166504) and control siRNA (sc-37007) targeting a scrambled sequence were purchased from Santa Cruz Biotechnology.

### 4.3. Immunofluorescence Staining

For indirect immunofluorescence staining, the hAD-MSCs cultured on Lab-Tek chamber slides (Nunc, Naperville, USA) were fixed with precold methanol for 5 min and permeabilized with 0.2% TritonX-100 in PBS for 10 min. Then, the cells were blocked with 10% normal goat serum in PBS at room temperature for 30 min. After that, the cells were incubated with the primary antibodies at 37°C for 1 h. The cells were washed with PBS twice and were then incubated with appropriate fluorescein isothiocyanate-conjugated secondary antibodies (Zhongshan Biotechnology Co., Beijing, China) for 30 min. Finally, the cells were stained with 4′6-diamidino-2-phenylindole (DAPI) and were mounted with a mounting solution (Vector Laboratories, Burlingame, USA) for observation under a fluorescence microscope (IX-71, Olympus, Tokyo, Japan).

### 4.4. Stimulation and Transfection

hAD-MSCs were seeded in six-well plates at a density of 5 × 10^5^ cells/well. After 24 h, the medium was replaced by a serum-free medium. After 2 h, the cells were stimulated with 5 *μ*g/mL poly(I:C)/LyoVec, HSV60/LyoVec, and LyoVec according to the manufacturer's instructions. For gene knockdown using siRNA, 2 × 10^5^ cells/well were seeded in six-well plates. After 24 h, the cells were transfected with 100 nM siRNA using 2 *μ*L Lipofectamine® RNAiMAX Reagent (Invitrogen, Carlsbad, USA). At 24 h after siRNA transfection, the cells were stimulated with poly(I:C)/LyoVec or HSV60/LyoVec.

### 4.5. Real-Time PCR

Total RNA was extracted using the Trizol reagent (Invitrogen) in accordance with the manufacturer's instructions. RNA was treated with RNase-free DNase I (Invitrogen) to remove genomic DNA contamination. RNA (1 *μ*g) was reversely transcribed into cDNA in 20 *μ*L reaction mixture containing 2.5 *μ*M random hexamer, 2 *μ*M deoxynucleotide triphosphate, and 200 U Moloney murine leukemia virus reverse transcriptase (Promega, Madison, WI, USA). Real-time PCR was performed using Power SYBR Green PCR Master Mix (Life Technologies, Foster City, USA) in an ABI PRISM 7300 real-time cycler (Life Technologies). Relative mRNA level of target genes normalizing to *β*-actin was given by 2^−ΔΔCt^, using the comparative threshold cycle method as described in Applied Biosystems.  Table 1 lists the sequences of primers.

### 4.6. Western Blot Analysis

The cells were lysed in ice-cold Radio Immunoprecipitation Assay (RIPA) lysis buffer (Beyotine, Nanjing, China) for 15 min. Equal amounts of protein (20 *μ*g) were separated on 10% SDS-PAGE gel and subsequently electrotransferred onto polyvinyl difluoride membranes (Millipore, Bedford, USA). The membranes were blocked in Tris-buffered saline (TBS, pH 7.4), containing 5% nonfat milk at room temperature for 1 h, and incubated with the primary antibodies and the appropriate horseradish peroxidase- (HRP-) conjugated secondary antibodies (Zhongshan Biotechnology Co., Beijing, China) at room temperature for 1 h. Antigen-antibody complexes were visualized using an enhanced chemiluminescence detection kit (Zhongshan Biotechnology Co.). *β*-Actin was used as loading control. The band intensities were quantified using ImageJ software (https://rsb.info.nih.gov/ij/).

### 4.7. Enzyme-Linked Immunosorbent Assay (ELISA)

The cells were cultured in six-well plates at a density of 5 × 10^5^ cells/well for 24 h and stimulated with poly(I:C)/LyoVec or HSV60/LyoVec. After 24 h, the cytokine levels in culture medium were measured using ELISA kits according to the manufacturer's instructions: IFN-*α* ELISA kit (BMS216, eBioscience, San Diego, USA), and IFN-*β* ELISA kit (KMC414101, Life Technologies).

### 4.8. Statistical Analysis

All data are presented as mean ± standard error of the mean (SEM). At least three independent experiments were performed, and each experiment was repeated twice. Student's *t*-test was used to determine significance between individual comparisons. The calculations were performed using SPSS version 11.0 statistic software. Statistical significance was defined as *P* value < 0.05.

## Figures and Tables

**Table 1:** Primers used for real-time PCR.

Targets genes	Primer pairs (5′ → 3′)	
	Forward	Reverse
GAPDH	GCACCGTCAAGGCTGAGAAC	ATGGTGGTGAAGACGCCAGT
TLR3	CTCAGAAGATTACCAGCCGCC	CCATTATGAGACAGATCTAATG
TLR7	TTAACCAATTGCTTCCGTGT	GGTGCCCACACtCAATCTG
TLR8	TGTGGTTGTTTTCTGGATTCAA	GCTCTCATGGCTTACATGA
MDA5	GTTGAAAAGGCTGGCTGAAAAC	TCGATAACTCCTGAACCACTG
RIG-I	GTGCAAAGCCTTGGCATGT	TGGCTTGGGATGTGGTCTACTC
TLR9	TGTGAAGCATCCTTCCCTGT	GAGAGACAGCGGGTGCAG
IFI16	ACTGAGTACAACAAAGCCATTTGA	TTGTGACATTGTCCTGTCCCCAC
DAI	CAACAACGGGAGGAAGACAT	TCATCTCATTGCTGTGTCCC
cGAS	CCTGCTGTAACACTTCTTAT	TTAGTCGTAGTTGCTTCCT
IFN-*α*	CTTGAAGGACAGACATGACTTTGG	GGATGGTTTCAGCCTTTTGGA
IFN-*β*	GCCGCATTGACCATCTATGAGA	GAGATCTTCAGTTTCGGAGGTAAC
Mx1	CAGCACCTGATGGCCTATCA	ACGTCTGGAGCATGAAGAACTG
OAS1	AGAGACTTCCTGAAGCAGCG	GAGCTCCAGGGCATACTGAG
ISG15	GAGAGGCAGCGAACTCATCT	CTTCAGCTCTGACACCGACA
